# Book Reviews

**Published:** 1901-11

**Authors:** 


HARRINGTON'S PRACTICAL HYGIENE. For Students and Practitioners of Medicine and Medical Officers. By CHARLES HARRINGTON, M. D., Assistant Professor of Hygiene in Harvard Medical School, Boston. In one very handsome octavo volume of 718 pages, with 105 engravings and 12 full-page plates in colors and monochrome. Philadelphia and New York: Lea Brothers & Co. 1901. [Cloth, $4.25, net.]

This work treats of a subject of great practical importance not only to physicians but to every person of intelligence. It is, moreover, one in which great changes and advances have been made within the last few years. We believe it is a subject inadequately taught in most medical schools. Improvements, however, in this direction can be stimulated by state medical examining boards. These latter should institute rigid examinations in hygiene, sanitation and prophylaxis. It is highly important that the young physician shall be well equipped in everything pertaining to the science and art of conserving the health, energy and welfare of the individual as well as the community.

Harrington has prepared a book essentially for the student—one which deserves to be well studied by undergraduates. It is impossible to compass hygiene in its entire domain in a single volume, hence later it will become essential to obtain larger works where certain topics of wider range can be found. But this volume is a complete, practical, authoritative exposition of the subject as it now exists and should be eagerly sought by every student in medicine.

---

PULMONARY CONSUMPTION, PNEUMONIA AND ALLIED DISEASES OF THE LUNGS. Their Etiology, Pathology and Treatment, with a chapter on Physical Diagnosis. By THOMAS J. MAYS, A. M., M. D., Professor of Diseases of the Chest in the Philadelphia Polyclinic: Visiting Physician to Rush Hospital for Consumption. Octavo, pages, 539. Illustrated. New York: E. B. Treat & Company. 1901. [Price, $3.00.]

This author is well known for his studies of pulmonary affections, through his published essays. He says that the underlying cause of this book is that he has conceived in the last thirty years some ideas that run counter to accepted teachings. He believes that pulmonary phthisis is generally a neurosis primarily, and that disintegration is secondary; that agents, influences, or conditions which jeopardise the integrity of the nervous system tend to develop pulmonary disease, especially phthisis; that remedies to be effective must act through the nervous system; that the counter-irritant action of nitrate of silver hypodermatically administered over the vagi in the neck is of special value; and that acute pneumonia and other forms of acute pulmonary disease are closely affiliated with disorder of the nervous system.

How well these views of cause, pathology, and cure are sustained must be determined by a careful reading of the author's pages. At all events, some interesting facts are presented and the book is a contribution of value to the study of pulmonary diseases and especially consumption.

---

PRINCIPLES OF SURGERY. By N. SENN, M. D., Ph. D., LL. D., Professor of Surgery in Rush Medical College in Affiliation with the University of Chicago; Professorial Lecturer on Military Surgery in the University of Chicago; Attending Surgeon to the Presbyterian Hospital; Surgeon-in-chief to St. Joseph s Hospital; Surgeon-General of Illinois. Third edition, thoroughly revised, with 230 wood-engravings, half-tones and colored illustrations. Royal octavo, pages, xiv.—700. Philadelphia: F. A. Davis Co. 1901. [Extra cloth, $4.50 net; sheep or half-russia, $5.50 net.]

The first appearance of this work, eleven years ago, marked an epoch in surgical literature. The author has been for so long a time a teacher of prominence and a voluminous author that whatever he says is entitled to weight. The student of medicine should become well grounded in the principles of surgery before undertaking to acquire a technical knowledge of the practice of the art. He must become familiar with the processes of bone repair before attempting to adjust and dress a fractured femur.

This third edition has been very thoroughly revised in its text while many additions have been made. Two new chapters, one on "Degeneration," and the other on "Blastomycetic Dermatitis," have been added, together with many new illustrations, most of the latter being original. It goes without saying that this edition will receive the same favor that was accorded the others. It is a standard work that should be found in every surgeon's library.

---

LECTURES UPON THE PRINCIPLES OF SURGERY. Delivered at the University of Michigan. By CHARLES B. NANCREDE, A. M., M. D., LL. D., Professor of Surgery and Clinical Surgery; Emeritus Professor of General and Orthopedic Surgery, Philadelphia Polyclinic, etc., etc. With an appendix containing a résumé of the principal views held concerning inflammation. By WILLIAM A. SPITZLEY, A B., M. D., Senior Assistant in Surgery, University of Michigan. Illustrated. Philadelphia: W. B. Saunders. 1899. [Price $2.50.]

This author has presented to surgeons the principles of the art in most readable and accessible form,—well adapted to the needs of the undergraduate.

Nancrede has discussed the important points pertaining to surgical principles, such as inflammation, surgical fever, hemorrhage and hemostasis, with great clearness. There are many other questions connected with the subject that it would be interesting to discourse upon, but in this somewhat belated notice it will not be expedient or necessary to do so. By some mischance the work escaped review at the proper time, and we can only say we very much regret that so good a book should have been overlooked. We now must content ourselves with saying that Nancrede has made an admirable contribution to the literature of present-day surgery, which is saying a good deal in these days of good surgical treatises.

A Practical Treatise on Simple and Chronic Specific Urethritis. By J. Henry Dowd, M. D , late Genito-Urinary Surgeon at the Buffalo Hospital of the Sisters of Charity: formerly Chairman of the Surgical Section of the Buffalo Academy of Medicine. Illustrated 16mo, pp. xxxi.—135. Buffalo: A. W. Landsettle. 1901. [Price, $1.50.]

In this excellent monograph the author has rendered an excellent service to every physician interested in the study or cure of gonorrheal infection. He has placed within easy reach of every such physician, a compact and scientific grouping of essential facts pertaining to the diagnosis and treatment of gonorrhea. Of special importance is it to know when the infection ceases to be virulent or when it can no longer be conveyed. Dr. Dowd has contributed much to make this greatly misunderstood part of the subject clearer, and we hope his useful book will be read by every physician who is called upon to treat the diseases with which it deals.

---

INTRODUCTION TO THE DIFFERENTIAL DIAGNOSIS OF THE SEPARATE FORMS OF GALLSTONE DISEASE; based upon his own experience gained in 433 laparatomies for gallstone. By PROFESSOR HANS KEHR, Helbersadt Authorised translation by William Wotkyns Seymour, A. B. (Yale), M. D. (Harvard); formerly Professor of Gynecology in the University of Vermont; Fellow of the American Association of Obstetricians and Gynecologists; Surgeon to the Samaritan Hospital, Troy. Octavo, pages 359. Philadelphia: P. Blakiston's Son & Co. 1901. [Price, $2.50.]

The surgery of biliary tract, of comparatively recent adoption as an accepted procedure in gall-stone disease, has made a wonderful record in the lives it has saved and the relief it has brought to mankind. The translator of this valuable work, himself, has been cured of the disease by cholecystotomy, at the hands of Mr. Lawson Tait, and has made over thirty operations for its relief in others.

The author has done more gall-stone operations than any other living surgeon, and his success has been remarkable—3.3 mortality in 422 operations. It is easily undeistood how such a record confers *ex. catherdra* authority upon the operator, who becomes more certain of his technique with each succeeding operation.

This is one of the most valuable recent contributions to surgical literature; it is one of the really and importantly good books to issue, in the midst of many indifferent ones, and will attract the attention of every physician or surgeon who treats gall-stone disease.

---

A TEXT-BOOK OF THE PRACTICE OF MEDICINE. By DR. HERMAN EICHHORST, Professor of Special Pathology and Therapeutics and Director of the Medical Clinic in the University of Zurich. Translated and edited by Augustus A. Eshner, M.D., Professor of Clinical Medicine in the Philadelphia Polyclinic. Two octavo volumes of over 600 pages each; over 150 illustrations. Philadelphia and London: W. B. Saunders & Co. 1901. [Price, per set, cloth, $6.00 net.]

A book on medicine from the German is always noted for its accuracy of detail. Eichhorst's treatise is no exception to this established rule. The translation by Eshner, is good rendering into smooth English of concise text, with such additions as are required to adapt it to our needs. The articles on the skin, venereal diseases, impotence and sterility in the male, and spermatorrhea are added to the usual contents of a work on practice, and will be found of great value to the general physician.

The classification employed by this author is excellent and the display type of heads and subheads is attractive to the eye. A number of good illustrations, some by the author and others by the translator, serve to explain the text here and there with clearness, while the publication of the work in two volumes adds to its convenience. It will be regarded by every student of internal medicine as a distinct addition to our literary resources, and is welcomed not only on that account, but because of its intrinsic merit as a guide to the diagnosis and treatment of disease.

---

Contemporary Celebrities from the album Mariani of Paris, France. New York: Published by Mariani & Co., 52 West 15th Street. 1901.

This beautiful collection of portraits is a credit to Mr. Mariani, and is artistically one of the choicest of its kind. It contains the pictures of many well-known personages, representing royalty, composers, authors, academicians, professional men, army and navy officers, artists and other celebrities. They are well executed wood-cut engravings, printed on heavy paper, and the album constitutes a souvenir of great value, which attests the popularity of Mr. Mariani and his wine of coca or "Vin Marani," as it is most appropriately named.

---

Transactions of the Southern Surgical and Gynecological Association. Volume XIII. Thirteenth annual session, held at Atlanta, Ga., November 12, 14 and 15, 1901. Secretary, Dr. W. D. Haggard, Jr., Nashville. Philadelphia: W. J. Dornan, Printer. 1901.

This splendid volume is replete with papers full of scientific interest relating to general surgery and gynecology. Several of the contributions are well illustrated and the discussions are all that could be desired to elaborate and strengthen the several points made by the authors.

If there were any doubts in the minds of the Fellows of the association as to the propriety of publishing its transactions in permanent form, a careful examination of this book would at once dispel them. The preparation of the volume exhibits great care on the part of the accomplished secretary, and it will prove a stimulus to further progressive work on the lines laid down by the founders of the association.

---

Transactions of the Mississippi Valley Medical Association. Twenty-sixth annual session, held at Asheville, N. C, October 9, 10 and 11, 1900. Volume II. Henry E. Tuley, M. D., Secretary.

This handsome volume is the second one to issue from this active association, although it has been organised for 26 years. It is largely due to the energy of Dr. Tuley, the present efficient secretary, that the association was induced to publish its work in an annual volume. The wisdom of this action is apparent to every experienced physician. While the publication of the proceedings and the papers in the journals is proper, yet these become scattered and in a few months are unavailable; whereas, in a compact and well-edited volume like the one at hand, reference can be made in a moment to the fact in question. It is doubtful policy for a medical society to publish a journal, but wise for it to issue such a volume of transactions as this one, and we congratulate the secretary and all concerned upon the success in this direction that has been reached.

---

The Johns Hopkins Hospital Reports. Volume X., Nos. 1 and 2. Baltimore: The Johns Hopkins Press. 1901.

The contents of this repoit will prove of great interest to microscopists and bacteriologists. The structure of malarial parasites is just now a study of importance; the bacteriology of cystitis, pyelitis, and pyelonephritis in women, is also important; and the rare report on the infection with strongyloides intestinalis, all of which are herein considered, will entitle the brochure to the favor of those who are pursuing studies in these subjects.

